# Depression Mediates the Association Between Childhood Emotional Abuse and the Onset of Type 2 Diabetes: Findings From German Multi-Cohort Prospective Studies

**DOI:** 10.3389/fpsyt.2022.825678

**Published:** 2022-04-06

**Authors:** Seryan Atasoy, Hamimatunnisa Johar, Toni Fleischer, Manfred Beutel, Harald Binder, Elmar Braehler, Georg Schomerus, Daniela Zöller, Johannes Kruse, Karl-Heinz Ladwig

**Affiliations:** ^1^Department of Psychosomatic Medicine and Psychotherapy, University of Giessen and Marburg, Giessen, Germany; ^2^Department of Psychosomatic Medicine and Psychotherapy, Klinikum Rechts der Isar, Technical University of Munich, Munich, Germany; ^3^Institute of Epidemiology, Helmholtz Zentrum München, Munich, Germany; ^4^Department of Psychiatry, Medical Faculty, Leipzig University, Leipzig, Germany; ^5^Department of Psychosomatic Medicine and Psychotherapy, University Medical Center of the Johannes Gutenberg University of Mainz, Mainz, Germany; ^6^Institute of Medical Biometry and Statistics, Faculty of Medicine and Medical Center, University of Freiburg, Freiburg im Breisgau, Germany; ^7^German Center for Diabetes Research (DZD), Munich, Germany

**Keywords:** depression, childhood emotional abuse, type 2 diabetes, epidemiology, longitudinal

## Abstract

**Background:**

The dysregulation of glucose homeostasis *via* mental health stress is increasingly acknowledged, whereby depression independently increases the risk of the onset of type 2 diabetes by up to 60%. Contributing mental health factors starting in early life have further been considered, indicating that exposure to childhood emotional abuse is associated with both depression and an increased onset of type 2 diabetes in adulthood. However, the potential role of depression within the emotional abuse and type 2 diabetes link remains unknown.

**Methods:**

Data were derived from community-dwelling participants in southern and northeastern Germany who participated in the longitudinal KORA-F4 and SHIP-3 studies. Multivariable logistic regression analyses adjusted for lifestyle, somatic, and psychological risk factors were used to investigate the association between childhood emotional abuse, assessed retrospectively by the *Childhood Trauma Screener*, and newly diagnosed type 2 diabetes cases, which were confirmed using a standard oral glucose tolerance test. The mediating role of depressive symptoms between childhood emotional abuse and type 2 diabetes was assessed by the *Patient Health Questionnaire-9* and calculated by using the Sobel test for mediation.

**Results:**

A total of 2,973 (53.2% women, 46.8% men) participants with a mean age of 49.7 were included in the analyses, of whom 5.9% (7.1% women, 4.5% men) reported emotional abuse in childhood. Participants exposed to childhood emotional abuse had a 1.70 (1.12–2.56; *p* = 0.02) times higher odds of depression in the fully adjusted model than unexposed participants. During the 6.5-year follow-up period, 104 (3.5%) participants developed type 2 diabetes. Participants who were exposed to childhood emotional abuse had a 2.56 (1.31–4.98, *p* = 0.005) times higher odds of developing type 2 diabetes than unexposed participants. This association was significantly mediated by the increased odds of depression in participants with childhood emotional abuse (Sobel Test, 1.84, *p* = 0.06; Goodman Test, 1.91, *p* = 0.05).

**Conclusion:**

The current results indicate that the increased likelihood of type 2 diabetes onset in participants who were exposed to childhood emotional abuse is significantly attributed to increased depression in adulthood.

## Introduction

As the incidence of type 2 diabetes continues to rise, better understanding of modifiable risk factors beyond the traditional determinants becomes imperative (1). The past few decades have seen increasing acknowledgment of mental health risk factors as predictors of type 2 diabetes. Among these, depression continues to be the most researched factor in the field. It is now well-acknowledged that depression is an independent risk factor for type 2 diabetes, increasing incidence by up to 60% in both clinical populations and individuals with elevated symptoms of depression (2–4). Additionally, the effect of depression remains even after controlling for established risk factors of type 2 diabetes, including body mass index, family history of diabetes, physical inactivity, diet, smoking, and alcohol consumption.

At the same time, a growing body of research has delved into contributing risk factors starting in early life (5). Adversity during childhood, in the form of childhood maltreatment including emotional, physical, and sexual abuse or neglect, disrupts normative developmental processes and magnifies risk for cardiometabolic health consequences later in life (6, 7). In a recent population-based retrospective study, 80,657 individuals who were exposed to childhood maltreatment had a 2.13 (1.86–2.45) times higher likelihood to develop type 2 diabetes in comparison to unexposed individuals (8). This link is further supported by meta-analytic evidence including 87,251 participants and 5,879 cases of incident type 2 diabetes, showing that childhood adversities can increase the onset of type 2 diabetes up to an odds ratio of 1.32 (1.16–1.51) (9).

Thus far, there is preliminary evidence that childhood adversity and increased incidence of type 2 diabetes may be mediated by the presence of depression in individuals who were exposed to childhood maltreatment (5). However, this line of evidence has not assessed childhood abuse (emotional or sexual) even though exposure to childhood emotional abuse—affecting up to 363 in 1,000 individuals worldwide (10)—is particularly related to the onset and course of depression (11–15). For instance, a recent meta-analytic review of 192 studies, including 68,830 participants, has demonstrated that emotional abuse in childhood has the strongest association of any form of childhood maltreatment in predicting depression in adulthood (13). Consequently, focused investigation of childhood emotional abuse, depression, and type 2 diabetes is necessary for improved understanding of the role of depression as the linking mechanism between childhood maltreatment and type 2 diabetes.

In the current study, we aim to shed light on the mediating role of depression in the link between childhood emotional abuse and incident type 2 diabetes among German community-dwelling participants from a multi-cohort study (16). We hypothesize that the predictive value of childhood emotional abuse on the increased risk of type 2 diabetes in adulthood can largely be attributed to detrimental effects of depression (5).

## Methods

The GESA consortium (GEnder-Sensitive Analyses of mental health trajectories and implications for prevention: A multi-cohort consortium) included high-quality data on mental and somatic symptoms in over 40,000 participants from multiple longitudinal cohorts in Germany (16): the Gutenberg Health Study (GHS) conducted in southeast Germany (17), the Cooperative Health Research in the Augsburg Region (KORA) conducted in southern Germany (18, 19), and the Study of Health in Pomerania (SHIP) conducted in northeast Germany (20). The combined samples originate from different German regions; their different socioeconomic characteristics will inform gender-sensitive analyses (16).

In the present investigation, data were derived from a total of 4,175 participants [2,270 participants from the KORA F4 (2006–2012) and 1,905 participants from the SHIP 3 (2008–2012) cohorts]. As the GHS cohort did not include data on childhood adversities, it has not been included in the present study (*N* = 15,010). Furthermore, exclusion of participants with missing follow-up data (*N* = 19), missing data on diabetes status at baseline (*N* = 238), and missing data on covariates (*N* = 945) has led to a pooled data composed of 2,973 participants. Missing data analyses revealed that participants with missing data were more likely to be older and have lower education than participants included in the current study (*p* < 0.001).

The study was carried out in accordance with the Declaration of Helsinki, including written informed consent of all study participants. The cohorts were approved by the ethics committees of the Statutory Physician Board of Rhineland-Palatinate (837.020.07; GHS), the University of Greifswald (BB 39/08; SHIP), and the Bavarian Chamber of Physicians (06068; KORA).

### Data Harmonization and Handling

The different measures, waves, and age structures of the cohorts require substantial harmonization and complicate interpretations (16). DataSHIELD (Data Aggregation through Anonymous Summary-statistics from Harmonized Individual LevEL Databases) was used to conduct a pooled analysis of multiple cohort study data, which enables describing and analyzing large-scale and complex interactions in epidemiological studies. Usually, sharing the individual-level data necessary for many epidemiological analyses raises concerns about privacy, particularly in sensitive topics (e.g., drinking habits, social status, and diseases). In DataSHIELD, only non-disclosive summary statistics are shared across sites, and specific individual data remain on local servers and thus inaccessible for all users (21). Methods with the potential to distinguish individual data (e.g., scatter plots, outliers, or extreme values identification) are prohibited in DataSHIELD, resulting in more limited statistical functionality than the standard case.

### Outcome: Type 2 Diabetes

Type 2 diabetes incidence was obtained after a mean of 6.5 years follow-up in KORA F4 and 5 years of follow-up in the SHIP-3 studies based on physician validated self-report, current use of glucose-lowering agents, and on a standard 75-g oral glucose tolerance test (OGTT). Glucose tolerance categories were defined using fasting and 2-h glucose levels according to WHO diagnostic criteria (22): normoglycemia (fasting glucose < 6.1 mmol/L and 2-h glucose < 7.8 mmol/L), prediabetes [impaired fasting glucose (IFG) (fasting glucose ≥ 6.1 mmol/L but < 7.0 mmol/L, and 2-h glucose < 7.8 mmol/L), impaired glucose tolerance (IGT) (fasting glucose < 6.1 mmol/L and 2-h glucose ≥ 7.8 mmol/L but < 11.1 mmol/L), or combined IFG/IGT], and newly diagnosed diabetes (fasting glucose ≥ 7.0 mmol/L or 2-h glucose ≥ 11.1 mmol/L) (22).

### Childhood Emotional Abuse and Additional Childhood Maltreatment Categories

Childhood emotional abuse was assessed by the 5-item Childhood Trauma Screener (23), which shows good sensitivity and specificity for the German version of the Childhood Trauma Questionnaire (CTQ) including 28 items (25 clinical items and 3 validity items) (24, 25). The remaining four items were used to assess the following trauma categories: emotional neglect, physical neglect, physical abuse, and sexual abuse. The severity of each childhood trauma category ranged from (1) *never experienced the specific trauma* to (5) *very often* experienced the specific trauma, with a sum score ranging from 5 to 25. Given the low number of participants with frequent trauma experience, we transformed each item into a binary variable (26). The threshold for the two neglect categories was the subscale (4) “*often*,” while for the two abuse items, it started with (3) “*occasionally*.” Sexual abuse had the strongest threshold, starting with (2) *“rare”* (23).

### Depression

Depressive symptoms were assessed by the 9-item Patient Health Questionnaire (PHQ-9), which is a validated and reliable screening instrument for assessing the severity of depressive symptoms in a primary care setting (27). The PHQ-9 scores the severity of each of the 9 diagnostic criteria for major depressive symptoms in the fourth edition of the Diagnostic and Statistical Manual of Mental Disorders (DSM-IV) (28) as “0” (not at all) to “3” (nearly every day) in the previous 2 weeks. The scores ranged from 0 to 27 and were dichotomized by using a total score cutoff ≥9 for severe depressive symptoms and <9 for mild or no depressive symptoms.

### Covariates

*Sociodemographic factors* were self-reported and included age, gender, years of education, and living arrangement (living alone vs. living with someone). *Lifestyle and clinical factors* include smoking, alcohol consumption, physical activity, parental history of type 2 diabetes body mass index, blood pressure, and low-density lipoprotein (LDL) cholesterol. Participants were classified as smokers when they reported that they currently smoke at least one cigarette per day. To assess physical inactivity, participants were classified as “physically inactive” during leisure time if they did not regularly participate in sports and if they were not active for at least 1 h per week in summer and winter. Alcohol consumption was classified into three categories: no alcohol consumption (0 g/day), moderate alcohol consumption (0.1–39.9 for men and 0.1–19.9 for women), and heavy alcohol consumption (≥40 g/day for men and ≥ 20 g/day for women) (29, 30). Parental history of type 2 diabetes was based on participants’ self-report of the type 2 diabetes in the father, mother, or both. Body height and body weight were determined by trained medical staff following a standardized protocol. Body mass index was calculated as weight in kilograms divided by height in square meters. Anxiety was assessed by dichotomization based on the cutoff ≥3 of the total score using the Generalized Anxiety Disorder (GAD)-2 instrument (31).

### Statistical Analysis

Analyses were performed in DataSHIELD version 4.1. DataSHIELD is a software program that contains several R packages, based on R-version 3.5.2, and can be applied to perform analyses while preserving data protection rights of sensitive research data.

We performed gender-specific descriptive analyses of the pooled sample. The descriptive data of sociodemographic, lifestyle, clinical, and mental health factors were obtained according to sex. Participant’s characteristics are presented as proportions or as mean ± standard deviation (SD), accordingly. Bivariate associations between groups were tested using the χ^2^ test for categorical variables and generalized linear regression (GLM) models for continuous variables.

In the pooled populations, multiple generalized linear regression models were employed to calculate β estimates, standard error (SE), and *p*-values for the associations between childhood emotional abuse and type 2 diabetes with different steps of adjustments. All models were adjusted for sociodemographic, lifestyle, cardiometabolic, and psychosocial risk factors. Multiple generalized linear regression models were performed to consider the interaction effect of gender and childhood neglect on type 2 diabetes incidence by including the gender by emotional and physical neglect (loneliness × gender) interaction term in the models.

Mediation analysis was performed to determine the potential mediating effect of depression on the association between childhood emotional abuse and type 2 diabetes, and was assessed by the Sobel test (32). To this end, the association between depression (mediator variable) and childhood neglect (independent variable), as well as the association between depression and type 2 diabetes (dependent variable) was tested in multiple generalized linear regression models.

Deviance residuals were examined to evaluate the model’s goodness of fit. The increase in deviance is evidence of a significant lack of fit.

## Results

A total of 2,973 participants (53.2% women, 46.8% men) with a mean age of 49.7 were pooled from the two study cohorts. As shown in Table 1, 5.9% (7.1% women, 4.5% men) of participants experienced childhood emotional abuse. At baseline, women were more likely to be younger and non-smokers, less likely to consume alcohol, and have lower BMI and blood pressure. However, women concurrently experienced more mental health impairment than men—they were significantly more depressed and anxious, and suffered from higher emotional and sexual abuse during childhood.

**TABLE 1:** Gender-specific characteristics of the pooled study population (*N*, % or mean ± SD) in 2,973 participants (1,583 women and 1,390 men).

Baseline characteristics	Total *N* = 2,973	Women *n* = 1,583	Men *n* = 1,390	*p*
**Sociodemographic factors**				
Age (±SD)	49.7	49.2 (11.4)	50.4 (11.8)	**0.004**
Education (years)	12.4	12.2 (2.4)	12.6 (2.6)	**<0.0001**
Lives alone	404 (13.6)	221 (14.0)	183 (13.1)	0.56
**Lifestyle factors**				
Smoking				**<0.0001**
Non-smoker	1,326 (44.6)	836 (52.8)	490 (35.2)	
Quit smoking	989 (33.3)	408 (25.8)	581 (41.8)	
Smokes irregularly	98 (3.3)	58 (3.7)	49 (2.9)	
Smokes regularly	560 (18.8)	281 (17.7)	279 (20.1)	
Alcohol consumption				**<0.0001**
Low	417 (14.0)	275 (17.4)	142 (10.2)	
Average	2,077 (69.9)	1,203 (76.0)	874 (62.9)	
High	479 (16.1)	105 (6.6)	374 (27.0)	
**Clinical factors**				
Parental history of T2D^a^	741 (24.9)	423 (26.7)	318 (22.9)	**0.05**
BMI (SD)	27.0	26.4 (5.0)	27.7 (4.0)	**<0.0001**
LDL-cholesterol (±SD)^b^	215.0	214.7 (42.3)	215.4 (40.0)	0.65
Systolic BP (±SD)	125.0	119.9 (17.7)	130.8 (16.6)	**<0.0001**
Diastolic BP (±SD)	79.2	76.9 (9.6)	81.8 (10.0)	**<0.0001**
**Mental health factors**				
Depression	329 (11.1)	231 (14.6)	98 (7.0)	**<0.0001**
Anxiety	416 (14.0)	280 (17.7)	136 (9.8)	**<0.0001**
Emotional abuse	176 (5.9)	113 (7.1)	63 (4.5)	**0.003**
Physical abuse	199 (6.1)	96 (7.4)	103 (6.7)	0.16
Emotional neglect	543 (18.3)	278 (17.6)	265 (19.1)	0.31
Physical neglect	851 (27.9)	441 (29.5)	410 (28.6)	0.35
Sexual abuse	157 (5.3)	135 (8.5)	22 (1.6)	**<0.0001**

*^a^Includes mother, father, or both parents.*

*T2D, type 2 diabetes.*

*^b^Low density lipo-protein.*

*p-values numbers marked in bold indicate numbers that are significant on the 95% confidence limit.

### Incidence of Type 2 Diabetes

**FIGURE 1:**
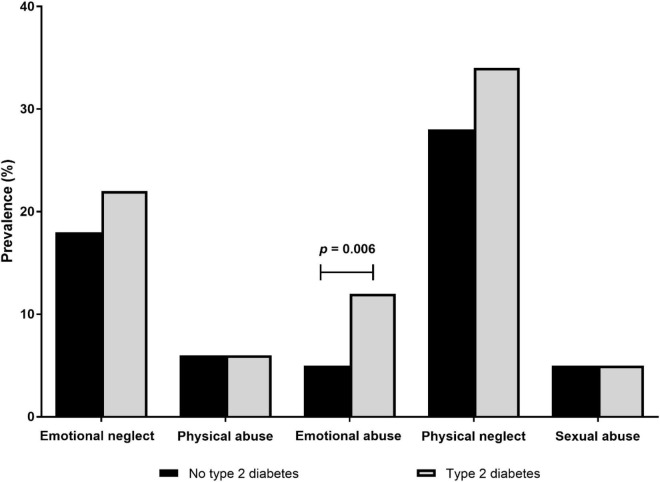
Cumulative incidence of type 2 diabetes in participants according to childhood maltreatment categories (*N* = 2,973).

During the follow-up period, there were 104 (3.5%) cases of type 2 diabetes incidence in the pooled cohorts. Despite the differences in baseline risk factors, women and men developed type 2 diabetes at a comparable rate during the follow-up period (3.3% of women and 3.7%, *p* = 0.56). However, as shown in Table 2, participants who developed type 2 diabetes were substantially older, were less educated, had worse lifestyle and cardiometabolic risk factors, and had worse depression in comparison to participants who did not develop type 2 diabetes. However, of the childhood maltreatment categories, although there was a general trend of higher type 2 diabetes incidence in those with history of childhood maltreatment, only childhood emotional abuse was significantly associated with type 2 diabetes (Figure 1).

**TABLE 2:** Characteristics of the pooled study population according to type 2 diabetes outcome (*N*, % or mean ± SD) in 2,973 participants (1,583 women and 1,390 men).

Baseline Characteristics	Total *N* = 2,973	No type 2 diabetes *n* = 2,869	Type 2 diabetes *n* = 104	*p*
**Sociodemographic factors**				
Women	1,583 (53.2)	1,531 (53.5)	52 (50.0)	0.56
Men	1,390 (46.8)	1,338 (46.6)	52 (50.0)	
Age (±SD)	49.7	49.5 (11.6)	56.8 (10.0)	**<0.0001**
Education (years)	12.4	12.4 (2.5)	11.6 (2.4)	**0.001**
Lives alone	404 (13.6)	384 (13.4)	20 (19.2)	0.11
**Lifestyle factors**				**0.04**
Smoking				
Non-smoker	1,326 (44.6)	1,288 (44.9)	38 (36.5)	
Quit smoking	989 (33.3)	942 (32.8)	47 (45.2)	
Smokes irregularly	98 (3.3)	97 (3.4)	1 (0.9)	
Smokes regularly	560 (18.8)	542 (18.9)	18 (17.3)	
Alcohol consumption				0.46
Low	417 (14.0)	406 (14.2)	11 (10.6)	
Average	2,077 (69.9)	1,999 (69.7)	78 (75.0)	
High	479 (16.1)	464 (16.2)	15 (14.4)	
**Clinical factors**				
Parental history of T2D^a^	741 (24.9)	695 (24.2)	46 (44.2)	**<0.0001**
BMI (SD)	27.0	26.8 (4.4)	32.0 (5.0)	**<0.0001**
LDL-cholesterol (±SD)	215.0	214.8 (40.7)	219.2 (53.1)	0.28
Systolic BP (±SD)	125.0	124.5 (17.9)	139.3 (18.4)	**<0.0001**
Diastolic BP (±SD)	79.2	78.9 (10.0)	85.4 (11.6)	**<0.0001**
**Mental health factors**				
Depression	329 (11.1)	307 (10.7)	22 (21.2)	**0.001**
Anxiety	416 (14.0)	396 (13.8)	30 (19.2)	0.15
Emotional neglect	543 (18.3)	520 (18.1)	23 (22.1)	0.36
Physical abuse	199 (6.1)	192 (6.7)	7 (6.7)	10.0
Emotional abuse	176 (5.9)	163 (5.7)	13 (12.5)	**0.007**
Physical neglect	851 (27.9)	815 (28.4)	36 (34.6)	0.20
Sexual abuse	157 (5.3)	151 (5.3)	6 (5.8)	0.99

*^a^Includes mother, father, or both parents.*

*T2D, type 2 diabetes.*

*p-values numbers marked in bold indicate numbers that are significant on the 95% confidence limit.

### Associations Between Childhood Emotional Abuse and Type 2 Diabetes Incidence

As shown in Table 3, participants who suffered from childhood emotional abuse experienced 2.56 times higher odds of incident type 2 diabetes in comparison to participants without any experience of childhood emotional abuse, even following adjustment for sociodemographic, lifestyle, cardio-metabolic, and mental health risk factors [OR 2.56 (95% CI 1.31–4.98), *p* = 0.005]. However, there were no gender-specific interactions found in this analysis (*p* = 0.39); hence, further gender-specific analyses were not carried out.

**TABLE 3:** The association between childhood emotional abuse and type 2 diabetes onset in the cohort pooled population of 2,973 participants (odds ratios, 95% confidence intervals).

	Odds Ratio (95% CI)	*p*
Emotional abuse	2.56 (1.31–4.98)	**0.005**
**Covariates**		
Sex	1.05 (0.65–1.68)	0.82
Age	1.04 (1.02–1.06)	**<0.0001**
Education years	0.90 (0.83–0.97)	**0.02**
**Parental history of T2D**		
Father	2.39 (1.27–4.46)	**0.006**
Mother	2.27 (1.39–3.71)	**0.001**
Regular smoking	1.80 (0.96–3.37)	**0.06**
High alcohol consumption	1.25 (0.52–3.01)	0.62
BMI	1.16 (1.12–1.21)	**<0.0001**
Cholesterol	0.99 (0.99–1.00)	0.50
Blood pressure	1.01 (1.00–1.02)	**0.0004**
Depression	2.03 (1.19–3.45)	**0.008**

*T2D, type 2 diabetes; CI, confidence interval.*

*p-values numbers marked in bold indicate numbers that are significant on the 95% confidence limit.

### Mediation of Childhood Emotional Abuse and Type 2 Diabetes Incidence by Depression

**FIGURE 2:**
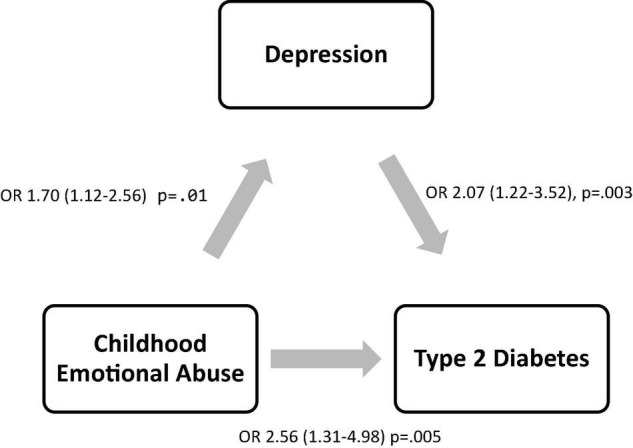
Mediation analysis of childhood emotional abuse, depression, and type 2 diabetes. Sobel test was used to test the significance of the effect of depression on the association between childhood emotional abuse and type 2 diabetes in fully adjusted regression analyses. Beta estimates with std error and *p*-values are reported.

To elucidate the pathway between the childhood emotional abuse–type 2 diabetes link, the role of depression as a mediator was analyzed (Figure 1). The conditions necessary to qualify depression as a mediation variable within this association were met (Figure 2). First, the findings confirmed that participants with childhood emotional abuse had a significantly higher risk of depression in adulthood in comparison to participants without childhood emotional abuse [OR 1.70 (95% CI 1.12–2.56), *p* = 0.01] when adjusted for sociodemographic, lifestyle, and clinical risk factors. Second, participants with depression had a significantly increased incidence of type 2 diabetes in comparison to participants without depression after full adjustment, excluding childhood emotional abuse [OR 2.07 (95% CI 1.22–3.52), *p* = 0.003]. Hence, mediation tests were used to determine whether depression influences the effect of childhood emotional abuse on the incidence of type 2 diabetes, showing a borderline significant Sobel Test (1.84, *p* = 0.06) and a significant Goodman Test (1.91, *p* = 0.05). These findings indicate that the pathway from childhood emotional abuse to the increased risk of type 2 diabetes is significantly associated with depression.

## Discussion

In the current multi-cohort investigation including 2,973 community-dwelling participants with a mean age of 49.7 years, nearly 6% of individuals experienced emotional abuse in his or her childhood. The prospective analyses herein confirmed the detrimental effects of childhood emotional abuse throughout individuals’ life span. Following adjustment for a comprehensive set of type 2 diabetes risk factors, childhood emotional abuse was independently associated with 1.70 higher odds of experiencing depression, as well as 2.56 times higher odds of developing type 2 diabetes in adulthood. However, further mediation analyses indicated that the substantial link between childhood emotional abuse and the increased onset of type 2 diabetes was indeed attributable to the high incidence of depression, extending the current understanding of the toxic effect of depression on type 2 diabetes.

All forms of childhood adversity reported herein were associated with an increased onset of type 2 diabetes (8); however, statistically significant results were only evident for participants who were specifically exposed to emotional abuse in childhood. This finding was in line with a previous study including a study of 14,493 young adults between 24 and 34 years old, which found a significant association between emotional abuse and incident type 2 diabetes—but only in women (33). Similarly, it has been shown that adults who experienced emotional abuse by their mother had significantly higher rates of self-reported diabetes than adults reporting no childhood maltreatment (OR = 3.4; 95% CI = 1.9–6.4) (34). Nevertheless, a recent meta-analysis on adverse childhood experiences and risk of type 2 diabetes did not overtly include the link between childhood emotional abuse and type 2 diabetes (9); hence, the detrimental effects of childhood emotional abuse on dysregulated glucose metabolism remain largely overlooked.

Emotional abuse affects the lives of millions of children worldwide (10) and significantly increases their risk of developing mental health conditions throughout the life span (13). The negative effects of childhood emotional abuse are so far reaching that among the childhood adversities, emotional abuse is most strongly associated with the onset and course of depression throughout the life span. It has recently been shown that exposure to emotional abuse in childhood increases the onset of depression by twofold in comparison to childhood physical abuse (14). Underlying reasons for the strong association between exposure to emotional abuse in childhood and depression in adulthood are attributed to a wide range of detrimental factors, including negative cognitive style (35), mentalizing incapacity (36), increased stress reactivity (37), negative self-referential processing (38), and low self-esteem (39). In contrast, a systematic review of the role of early life stress in adult psychiatric disorders has shown that physical neglect has the smallest effect size for the onset and severity of mental health conditions (40). Hence, the increased likelihood of depression following exposure to childhood emotional abuse is a significant pathway to consider in predicting and preventing future health conditions in these individuals.

It is well-established that depression can have detrimental outcomes, including mortality *via* increasing suicidality (41, 42). Similarly, depression is an independent risk factor for increased morbidity, such as its role in the increased onset of type 2 diabetes (2, 3). The pathophysiological factors associated with depression are largely comparable to the risk of established lifestyle and somatic risk factors of diabetes. Namely, depression—*via* the associated allostatic load (43, 44)—is thought to increase insulin resistance by dysregulation of the HPA axis (45). The strong effect of depression on type 2 diabetes is even evident in amplifying the effect of obesity for the risk of type 2 diabetes (46). Hence, the higher predisposition and prevalence of depression in adults who already experienced emotional abuse during childhood can have a significant amplification role in the association between emotional abuse and type 2 diabetes. This is in line with “two-hit” hypothesis in various mental health disorders, where emotional abuse in childhood can be considered the first hit, disrupting early central nervous system development, and priming the nervous system for an increased vulnerability to a second hit that may occur as depression later in life (47).

Furthermore, there were significant gender differences to consider in the experience of childhood maltreatment and additional mental health factors at baseline—women had substantially higher prevalence of emotional abuse and depression in comparison men (48), but men had increased lifestyle and clinical risk factors. Nevertheless, women and men developed type 2 diabetes at a comparable rate during the follow-up period, suggesting comparability between physical risk factors in men and mental health risk factors in women. In line with this, women have been shown to be at greater risk of early life adversity and affective disorders throughout life (49). Therefore, the continued focus for how sex differences in stress responses may predict disease risk and resiliency is critical for developing preventive strategies and treatments (49).

The strengths of the current study include its prospective study design, validated diagnosis of type 2 diabetes and OGTT at both time points, the measurement of childhood maltreatment and depression based on a highly specific and validated instrument, as well as comprehensive adjustments for sociodemographic, metabolic, and lifestyle factors. Additionally, the southern and northeastern regions of Germany included in the GESA consortium include varying levels of socioeconomic status and health, hence considerably representative of the general population. However, the association reported herein may be attenuated by confounders that were not available in the current study. Furthermore, participants with missing data were older and had lower levels of education; hence, the current study may underestimate true risk, as evident by the relatively low incidence of type 2 diabetes. Lastly, we cannot discern causality as individuals with depression might be more likely to remember emotional abuse in childhood.

In conclusion, the present investigation has added a new perspective to the toxic effects of childhood adversity, indicating that the association between childhood emotional abuse and the increased long-term risk of type 2 diabetes is significantly linked to and amplified by depression. These clinically relevant results once again emphasize the relevance of public health initiatives in targeting depression in primary care settings (50). However, more importantly, as depression has many facets and is likely to reach back to childhood adversities, public health initiatives must increasingly target at-risk populations as early as possible—in learning to identify, increase psychosocial support, and build resilience, the lifelong mental and physical health burden in individuals who experience early-life trauma may be significantly improved.

## Data Availability Statement

The data analyzed in this study is subject to the following licenses/restrictions: GESA is a multi-cohort project building on SHIP and KORA, where the data availability is limited to the local storage guidelines. Data access rights must be requested at each cohort and subsequently data access can be granted by the authors of this manuscript *via* DataSHIELD. Requests to access these datasets should be directed to MB, manfred.beutel@unimedizin-mainz.de.

## Ethics Statement

The studies involving human participants were reviewed and approved by the Statutory Physician Board of Rhineland-Palatinate (837.020.07; GHS), University of Greifswald (BB 39/08; SHIP), Bavarian Chamber of Physicians (06068; KORA). The patients/participants provided their written informed consent to participate in this study.

## Author Contributions

DZ, HJ, and HB organized the database. SA performed the statistical analysis and wrote the first draft of the manuscript. K-HL and JK wrote sections of the manuscript. All authors contributed to conception and design of the study, manuscript revision, read, and approved the submitted version.

## Conflict of Interest

The authors declare that the research was conducted in the absence of any commercial or financial relationships that could be construed as a potential conflict of interest.

## Publisher’s Note

All claims expressed in this article are solely those of the authors and do not necessarily represent those of their affiliated organizations, or those of the publisher, the editors and the reviewers. Any product that may be evaluated in this article, or claim that may be made by its manufacturer, is not guaranteed or endorsed by the publisher.
